# Microbiological Profiles and Resistance Patterns in Pediatrics With Cancer: An 8‐Year Study at a Comprehensive Cancer Center in Jordan

**DOI:** 10.1002/cnr2.70132

**Published:** 2025-03-10

**Authors:** Dana Hassouneh, Razan Zatarah, Aseel AbuSara, Lama Nazer, Amal Abu Ghosh, Haitham Al Aryan, Iyad Sultan

**Affiliations:** ^1^ Department of Pharmacy King Hussein Cancer Center Amman Jordan; ^2^ Department of Pediatrics King Hussein Cancer Center Amman Jordan; ^3^ Patient Journey and Health Information Management King Hussein Cancer Center Amman Jordan; ^4^ Faculty of Medicine University of Jordan Amman Jordan; ^5^ Artificial Intelligence and Data Innovation Office, King Hussein Cancer Center Amman Jordan

**Keywords:** bacteria, cancer, infection, multidrug resistant organisms, pediatrics

## Abstract

**Introduction:**

Infections impact morbidity and mortality in pediatric cancer patients, yet limited studies have assessed the microbiological profiles and susceptibility patterns of pathogenic bacteria in this population. This study aimed to investigate bacterial profiles and temporal resistance changes in pediatrics with cancer.

**Methods:**

We identified positive cultures between January 2015 and December 2022 for pediatric patients diagnosed with cancer at age < 18 years. Electronic records provided patient demographics, microbiological profiles and resistance patterns. Using “R” programming, the dataset was refined, selecting patients' first isolate within a 30‐day period, and categorizing strains based on multidrug‐resistant (MDR) and extensively drug‐resistant (XDR) predefined criteria. Additionally, we analyzed changes in resistance patterns over the study period.

**Results:**

Out of 1215 patients, 2992 bacterial isolates were reported, with 66% being Gram‐negative bacteria. Urine was the most common site of infection, representing 48% of cases. Among these, 42% were MDR and 2% XDR. MDR prevalence was 45% for *Escherichia coli* , 21% for *Klebsiella pneumonia*, and 14% for *Staphylococcus aureus* . *Acinetobacter baumannii* and *Pseudomonas aeruginosa* displayed XDR at 79% and 21%, respectively. *Methicillin‐resistant S. aureus* decreased from 71% in 2015 to 54% in 2022. MDR *Klebsiella pneumonia* peaked in 2021. MDR *Pseudomonas aeruginosa* decreased from 44% in 2015 to 16% in 2022.

**Conclusion:**

Drug resistance was detected in 50% of the isolates with most being Gram‐negative and MDR. Further research is needed to identify risk factors for resistance, aiming to refine empiric antimicrobial therapy.

**Abbreviations**
AMR: Antimicrobial resistance
CLSI: Clinical and Laboratory Standards Institute
CNS: Central Nervous System
CoNS: Coagulase‐Negative Staphylococcus
CSF: Cerebrospinal fluid
ESKAPE: *Enterococcus faecium, Staphylococcus aureus, Klebsiella pneumoniae, Acinetobacter baumannii, Pseudomonas aeruginosa*, and *Enterobacter spp*
EUCAST: European Committee on Antimicrobial Susceptibility Testing
KHCC: King Hussein Cancer Center
MDR: Multidrug‐resistant
MRSA: Methicillin‐Resistant *Staphylococcus Aureus*

PDR: Pandrug‐resistant
SD: Standard deviation
XDR: Extensively drug‐resistant

## 1 Introduction

Pediatrics with cancer are prone to a spectrum of bacterial, viral, and fungal infections due to their immune system suppression resulting from the disease itself or chemotherapy [1]. These infectious complications significantly increase morbidity and mortality within this group of patients [1]. In addition, the wide utilization of broad‐spectrum antimicrobials has contributed to the emergence of anti‐microbial resistant pathogens which may differ from those prevalent in the community or other healthcare settings.

In a 2019 prospective study by Garrido et al. [2], the epidemiology and microbiological characteristics of infections in pediatric cancer patients varied based on malignancy type and related factors. A six‐year retrospective study by Spychala et al. [1] highlighted bloodstream infections as the most common bacterial infections, with a prevalence of Gram‐negative bacteria. Furthermore, the study raised concerns about the increase in multi‐drug resistant (MDR) pathogens but did not explore temporal changes over the years. While previous studies have explored bacterial infections, patient characteristics, and outcomes, fewer studies have explored infections from various sites besides the bloodstream [1, 3, 4]. Additionally, the studies were limited by small sample sizes and relatively short time frames [3, 4, 5, 6].

The rise in multidrug‐resistant (MDR) bacteria worldwide poses a significant threat to public health, driven by the overuse of antibiotics, inadequate infection prevention measures, and insufficient antimicrobial stewardship programs. These factors create an environment where resistant bacteria can thrive and spread. However, when it comes to the high prevalence of MDR bacteria among pediatric cancer patients, the unique vulnerabilities of this population—such as immunosuppression from chemotherapy, frequent hospitalizations, and prolonged use of invasive devices—further exacerbate the issue.

Currently, scarcity of data exists that comprehensively describes the epidemiology of infections and the microbiological profiles of the causative agents in pediatrics with cancer, particularly in low‐ and middle‐income countries. Understanding the regional epidemiology is crucial to mitigate the impact of infections on such a vulnerable population. This highlights the importance of ongoing surveillance and data analysis in light of regional disparities.

Our primary outcome was to describe the microbiological profiles of bacterial pathogens isolated from positive cultures in pediatrics with cancer. This included identifying their type along with their resistance and susceptibility patterns. Our secondary outcomes were directed towards identifying temporal changes of MDR and extensively drug‐resistant (XDR) pathogens over the years. This shall serve as a guide to healthcare providers to better select empirical antimicrobials and aid in the development of infection prevention and control measures.

## 2 Methods

### 2.1 Study Setting and Design

This was a single‐center, retrospective cohort study of pediatrics with cancer treated at King Hussein Cancer Center (KHCC). The hospital provides comprehensive cancer care to pediatrics with cancer in the inpatient and outpatient settings. The Institutional Review Board at KHCC approved this study with a waiver of informed consent due to its retrospective nature (No. 23 KHCC 155).

### 2.2 Patients and Materials

Patients enrolled in our study were those aged < 18 years at diagnosis with cancer, with a positive bacterial culture obtained between January 1st, 2015 and December 31st, 2022. Cultures taken in the outpatient and inpatient settings were included. The electronic medical records were used to extract patients' demographics and microbiological profiles, which included age, gender, type of malignancy, site of specimen, type of microorganism, as well as their susceptibility and resistance towards various types of antibiotics. The microbiology results were based on local testing done at KHCC that abide by the Clinical and Laboratory Standards Institute (CLSI) guidelines.

Microorganism identification at KHCC involves patients undergoing sample collection from the blood, urine, respiratory, cerebrospinal fluid (CSF), as well as skin and wound cultures. These samples are then sent to the microbiology laboratory for assessment. Results that were associated with routine surveillance tests or screening specimens such as skin, throat, nasal, and perianal swabs were excluded.

Our database underwent data cleaning by utilization of the “R” programming language version 4.3.2 [7] that incorporates an Antimicrobial Resistance (AMR) package [8] designed to interface with multitudes of features related to pathogen types and antimicrobials. The AMR package is specifically designed to process data related to bacterial pathogens and to allow users to identify various taxonomic properties of microorganisms, such as classifying bacterial isolates as Gram‐positive or Gram‐negative based on their taxonomic classification.

Recognizing the potential for multiple positive cultures from a single patient within a given timeframe, a “first isolate” function was applied. This function was adjusted to a 30‐day duration to reflect an episode‐based isolate [9].

Furthermore, the AMR package was used to assess susceptibility and resistance patterns of identified pathogens, including the identification of MDR organisms, XDR organisms, and pandrug‐resistant (PDR) organisms based on definitions proposed by Magiorakos et al. [10]. According to these guidelines, MDR is defined as acquired non‐susceptibility to at least one agent in three or more antimicrobial categories. XDR is defined as non‐susceptibility to at least one agent in all but two or fewer antimicrobial categories, and PDR is defined as non‐susceptibility to all agents in all antimicrobial categories, applied to both Gram‐ positive and Gram‐ negative organisms. Susceptibility patterns of pathogens towards antibiotics were also analyzed based on the CLSI guidelines. Gram‐negative pathogens included *Acinetobacter baumanni*, *Escherichia coli* , *Klebsiella pneumonia*, and *Pseudomonas aeruginosa* . Susceptibility of Gram‐positive pathogens including *Staphylococcus aureus* , *Enterococcus faecalis* , *Enterococcus faecium* , *Viridans Group Streptococcus*, and *Streptococcus pneumonia* were assessed against antibiotics such as penicillin and vancomycin. Susceptibility results were interpreted as per the European Committee on Antimicrobial Susceptibility Testing (EUCAST) recommendations. A bacterial isolate was deemed to be non‐susceptible if it tested resistant or intermediate according to the criteria set by the EUCAST or CLSI as used by our center. We examined the annual resistance of the ESKAPE bacterial pathogens from 2015 to 2022. Those pathogens include the following: *E. faecium* , *S. aureus* , *K. pneumoniae* , *A. baumannii* , *P. aeruginosa* , and *Enterobacter spp*.

Possible contaminants were managed systematically. Microorganisms identified as potential contaminants were determined through a synthesis of relevant studies [11, 12, 13], *Propionebacterium, Corynebacterium, Bacillus*, and *Micrococcus* were consistently excluded across all infection sites. We considered potential contamination from the *Coagulase‐negative Staphylococci* (CoNS) family. For CoNS, the following was implemented: if a patient yielded 2 or more positive cultures for CoNS from the same site within a 3‐day period, it was considered a true positive infection and included; conversely, if only 1 positive CoNS culture was identified for the same patient on the same day at the same site, it was classified as a contaminant and excluded from analysis.

In our center, pediatric patients with cancer receive prophylactic antimicrobial agents tailored to their specific disease type and risk status. Patients with acute myeloid leukemia are started on prophylaxis at the onset of neutropenia. The regimen typically includes ciprofloxacin, vancomycin, and trimethoprim‐sulfamethoxazole. Pediatric patients with acute lymphoblastic leukemia receive trimethoprim‐sulfamethoxazole prophylaxis early in treatment. Nonetheless, prophylactic antimicrobials are also commonly used in pediatric patients with solid tumors or lymphomas, particularly those with underlying immunodeficiency.

We have implemented a targeted antimicrobial stewardship strategy in both inpatient and outpatient settings focusing on the use of certain antibiotics because of issues of cost, resistance, and toxicity. For select agents such as piperacillin/tazobactam, cefepime, amikacin, and meropenem, and certain anti‐microbial combinations, approval from infectious diseases or defined service consultants is required before prescription with documentation of the approval status. This ensures these antibiotics are used judiciously, with careful consideration of indication, dosing, and duration.

### 2.3 Statistical Analysis

Continuous data were presented as mean and standard deviation (SD) while nominal data were presented as numbers and percentages. Visualization tools, such as bar charts were utilized to illustrate the distribution of key parameters.

## 3 Results

### 3.1 Study Population Characteristics

In this study, we evaluated 1215 pediatric patients, most of whom were diagnosed with hematological malignancies which included leukemia's (44%) and lymphomas (11%). Followed by CNS tumors (13%), sarcomas (11%), while the remaining patients had other solid tumors (21%). The mean age of the population at diagnosis with cancer was 6.89 years (SD ± 5.77). The cohort comprised of 51% females.

### 3.2 Microbiological Profiles of Pathogens

Over the 8‐year study period, a total of 2992 episode‐based bacterial isolates were identified. The isolates were predominantly Gram‐negative (66%) with *E. coli* (23%), *K. pneumonia* (12%), and *P. aeruginosa* (10%) being the most frequently isolated pathogens. The Gram‐positive bacteria, which constituted 34% of the isolates, were primarily represented by *S. aureus* (9%), *Viridans Group Streptococcus* (6%), and *Coagulase‐negative Staphylococcus (CoNS)* (4.5%) spp.

### 3.3 Site of Infections and Pathogen Distribution

**FIGURE 1:**
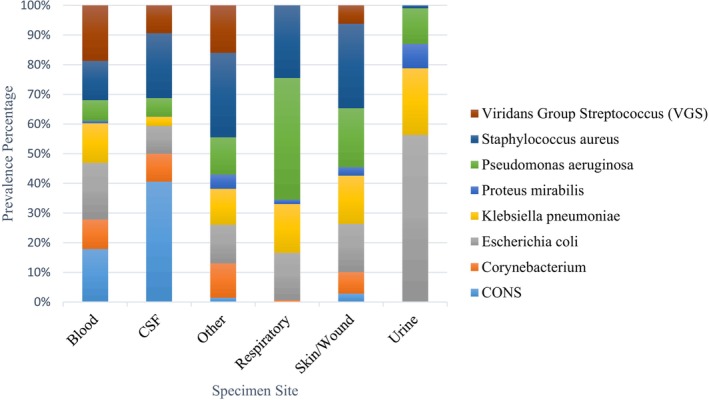
Distribution and prevalence of pathogens across different sites. Urine (41%) and bloodstream (36%) were the main infection sites, with *Escherichia coli* , Klebsiella pneumonia, and *Pseudomonas aeruginosa* prevalent in urine, and *Escherichia coli* , Viridans Group Streptococcus, and Coagulase‐negative Staphylococcus common in blood.

The primary site of infection was found to be urine (41%), followed by bloodstream (36%), skin & wound (13%), respiratory (8%), and CSF (2%). The most prevalent isolates in the urine were *E. coli* (56%), *K. pneumonia* (23%), *and* *P. aeruginosa* (12%). While those predominating in blood were *E. coli* (19%), *Viridans Group Streptococcus* (18.7%), and *Coagulase‐negative Staphylococcus* (17.9%). The distribution and prevalence of pathogens across different sites are shown in (Figure 1).

### 3.4 Antimicrobial Resistance Patterns

**FIGURE 2:**
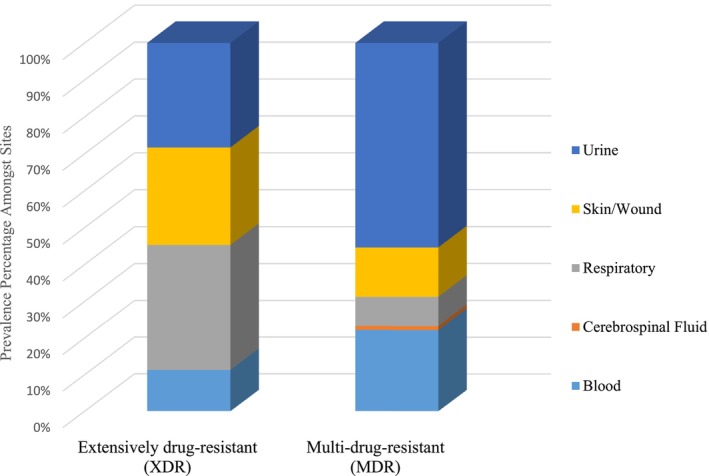
Distribution of resistant pathogens amongst sites. Of all isolates, 42% were MDR and 2% were XDR, with no PDR pathogens. MDR pathogens were primarily found in urine, while XDR pathogens were most commonly isolated from respiratory sites.

Among the bacterial isolates, 42% met the criteria for MDR and 2% were XDR pathogens. None of the pathogens were PDR. The highest prevalence of MDR pathogens was noted in *E. coli* (45%), *K. pneumonia* (21%), and *S. aureus* (14%). *Acinetobacter baumani* XDR isolates reached 79% while *P. aeruginosa* isolates exhibited XDR in 21% of the cases. The distribution of resistant pathogens amongst sites of infection are shown in (Figure 2).

### 3.5 Temporal Changes and Trends in Patterns of Resistance Between 2015 and 2022

**FIGURE 3:**
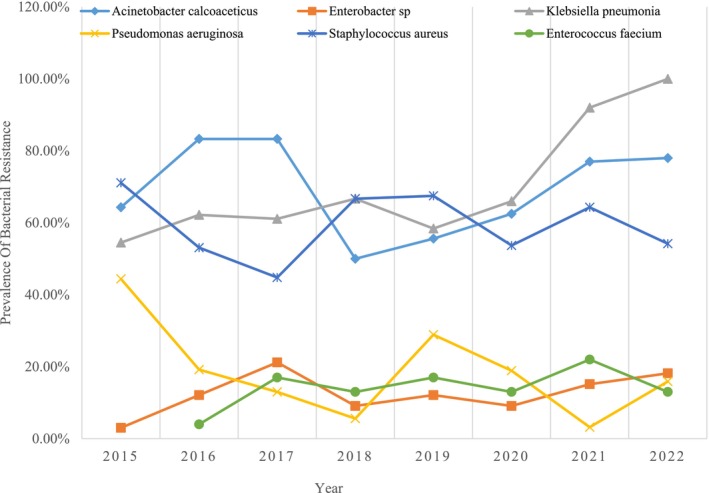
Annual resistance trends of ESKAPE pathogens from 2015 to 2022. Methicillin‐resistant *Staphylococcus aureus* (MRSA) incidence declined from 71% in 2015 to 54% in 2022. MDR *Pseudomonas aeruginosa* decreased from 44% in 2015 to 16% in 2022.

**FIGURE 4:**
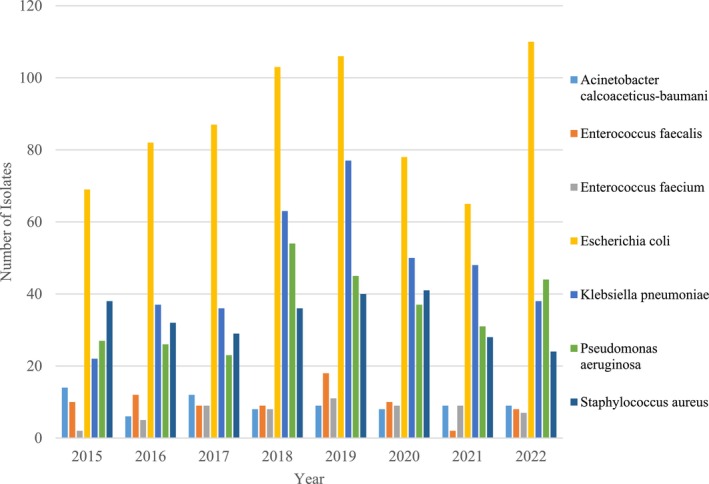
Number of select bacterial isolates per year from 2015 to 2022. Over time, *Escherichia coli* and *Klebsiella pneumoniae* increased, while *Acinetobacter‐baumani* and *Pseudomonas aeruginosa* declined. *Enterococcus faecalis* peaked in 2019, and *Staphylococcus aureus* remained relatively stable with minor fluctuations.

The annual resistance of the ESKAPE bacterial pathogens from 2015 to 2022 are presented in (Figure 3). *Methicillin‐resistant S. aureus* (MRSA) incidence decreased from 71% in 2015 to 54% in 2022. MDR *P. aeruginosa* showed a declining trend from 44% in 2015 to 16% in 2022 (Figure 4).

### 3.6 Susceptibility Towards Antibiotics

*K. pneumonia* showed good susceptibility to carbapenems with 100% of the isolates tested being susceptible to imipenem and meropenem. *E. coli* isolates demonstrated low susceptibility towards cefazolin, ceftriaxone, ceftazidime, and ciprofloxacin at susceptibility rates of 30%, 38%, 39%, and 54%, respectively. On the other hand, *P. aeruginosa* displayed high susceptibility towards meropenem (84%), piperacillin/tazobactam (89%), and amikacin (90%). *Enterobacter spp*. demonstrated a susceptibility of 68% towards cefepime, 88% towards amikacin, and 100% towards carbapenems (imipenem and meropenem). Only 32% of *A. baumanni* isolates were susceptible to carbapenems. Susceptibility of Gram‐negative pathogens towards antibiotics are presented in Table 1. Amongst Gram‐positive isolates, 100% of *E. faecalis* and *E. faecium* isolates displayed susceptibility towards vancomycin. Only 2% of the *S. aureus* isolates displayed susceptibility towards penicillin. Susceptibility of Gram‐positive pathogens towards antibiotics are presented in Table 2.

**TABLE 1:** Susceptibility of Gram‐negative pathogens towards antibiotics.

Antibiotic	Penicillins					Cephalosporins			
	Ampicillin	Ampicillin/sulbactam	Ticarcillin/clavulanate	Piperacillin	Piperacillin/Tazobactam	Cefazolin	Ceftriaxone	Ceftazidime	Cefepime
Bacteria									
*Acinetobacter baumannii*	2%	37%	36%	33%	35%	NA	21%	35%	35%
Enterobacter spp.	NA	1%	67%	49%	81%	NA	NA	NA	68%
*Escherichia coli*	8%	50%	70%	8%	87%	30%	38%	39%	56%
Klebsiella pneumonia	NA	51%	NA	NA	84%	34%	40%	40%	55%
*Pseudomonas aeruginosa*	NA	NA	69%	88%	89%	NA	NA	85%	88%

Antibiotic	Carbapenems			Quinolones		Aminogly‐cosides		Others		
	Ertapenem	Imipenem	Meropenem	Ciprofloxacin	Levofloxacin	Amikacin	Gentamicin	Aztreonam	Cotrimoxazole	Minocycline
Bacteria										
*Acinetobacter baumannii*	NA	32%	32%	31%	30%	35%	39%	NA	52%	52%
Enterobacter spp.	87%	100%	100%	74%	NA	88%	80%	61%	58%	NA
*Escherichia coli*	95%	100%	100%	54%	2%	98%	76%	40%	26%	NA
Klebsiella pneumonia	91%	100%	100%	69%	10%	94%	75%	42%	36%	NA
*Pseudomonas aeruginosa*	NA	82%	85%	88%	85%	90%	88%	82%	47%	100%

**TABLE 2:** Susceptibility of Gram‐positive pathogens towards antibiotics.

Antibiotic	Penicillin	Ampicillin	Oxacillin	Ceftriaxone	Erythromycin	Clindamycin	Cotrimoxazole	Levofloxacin	Vancomycin	Teicoplanin	Linezolid
Bacteria											
Coagulase‐negative Staphylococcus (CoNS)	5%	NA	28%	NA	18%	57%	59%	78%	NA	99%	100%
*Enterococcus faecalis*	90%	88%	NA	NA	NA	NA	NA	74%	100%	99%	100%
*Enterococcus faecium*	5%	10%	NA	NA	NA	NA	NA	30%	100%	68%	100%
*Staphylococcus aureus*	2%	NA	40%	NA	67%	75%	97%	94%	NA	100%	100%
*Streptococcus pneumoniae*	NA	NA	NA	NA	47%	72%	72%	100%	100%	NA	100%
Viridans Group Streptococcus (VGS)	NA	NA	NA	83%	34%	72%	NA	90%	100%	NA	100%

## 4 Discussion

In this study, we analyzed the microbiological profiles and resistance patterns of pathogenic bacteria in pediatrics with cancer. In addition, we evaluated the temporal changes in resistance patterns over an 8‐year period. Gram‐negative bacteria were the predominant organisms with the most common site of infection being urine. These results align with the results proposed by Spychala et al. [1] in which the most prevalent strain of pathogens were Gram‐negative bacteria. However, when looking at the most commonly prevalent Gram‐negative pathogens, our results are comparable to those obtained in a study by Kuo et al. [14] carried out on pediatrics with leukemia over a duration of 10 years in which the pre‐dominating pathogens were: *E. coli* (23%), *K. pneumonia* (12%), and *P. aeruginosa* (10%).

The most common Gram‐negative pathogens have been described in previous studies across regions in the middle east [15]. *E. coli* and *K. pneumoniae* were identified as the predominant Gram‐negative bacteria in hospital‐acquired infections among pediatric patients including those diagnosed with cancer under 18 years in the Egypt, Saudi Arabia, and Lebanon [15]. The similarity in pathogen distribution across different patient populations and geographical regions may stem from shared environmental exposures among pediatric patients. Studies suggest that exposures could include common sources of infection within healthcare settings, such as medical equipment, invasive procedures, or contact with healthcare personnel [15, 16]. Additionally, antimicrobial usage patterns, including the widespread use of prophylactic antibiotics or empirical treatment regimens, may exert selective pressure on bacterial populations, favoring the proliferation of certain strains over other.

At our center, antibiotics are dispensed based on physician prescriptions, possibly more frequently than average due to our immunocompromised patient population and heightened concerns about bacterial infections. Limited data is available on MDR pathogen infections in Middle Eastern pediatric oncology patients, as most existing studies focus on adults. The high prevalence of MDR bacteria in pediatric oncology patients is likely influenced by multiple factors. These include the intensive use of broad‐spectrum antibiotics for prophylaxis and treatment of infections during prolonged periods of immunosuppression, as well as the frequent hospital admissions. Additionally, high‐risk conditions such as febrile neutropenia necessitate the repeated use of empirical therapies with antimicrobial agents such as piperacillin/tazobactam, amikacin, cefepime, or meropenem, facilitating the emergence of resistance.

Gram‐negative pathogens are becoming more resistant to antibiotics worldwide and are notorious for their ability to develop resistance through different mechanisms [17]. The identification of MDR organisms and their prevalence, particularly in *E. coli* , *K. pneumoniae* , and *P. aeruginosa* , underscores the pressing concern of antimicrobial resistance in pediatric oncology. *P. aeruginosa* displayed a unique pattern; while some isolates showed susceptibility to antibiotics like piperacillin/tazobactam, amikacin, and meropenem, others were XDR. However, the prevalence of MDR *P. aeruginosa* decreased by 2022. We hypothesize that this decline could be due to adopting more prescribing restrictions on antimicrobials. In addition, we suggest the adoption of antimicrobial stewardship in pediatric oncology.

With respect to Gram‐positive pathogens, temporal shifts over 8 years have indicated a decreasing pattern in MRSA aligning with Diekema et al. [18] observations of a 20‐year trend in antibiotic susceptibility among *S. aureus* . Their global surveillance program revealed a decline in MRSA prevalence over the past decade despite it being considered a crucial pathogen with serious threat to public health. According to our results, prevalence of MRSA dropped by around 24% between the years 2015 and 2022. Other studies suggest that such change might occur due to the use of modalities such as antiseptic agents used for decolonization, in central lines care, or as oral rinses [19]. Dai et al. [20] conducted an epidemiologic investigation to determine the dynamic changes of *S. aureus* infections between 2008 and 2017. Their research found that the rate of respiratory infections dropped from 76% to 52% during the study period, leading to a notable decrease in MRSA cases. Additionally, they observed a significant reduction in the prevalence of a specific MRSA strain, which played a role in the overall decline of MRSA cases. These findings shed light on the intricate dynamics among *S. aureus* clones within healthcare settings.

We have focused on the notorious ESKAPE pathogens, known for their potential to evade antimicrobial treatment and to comprise the largest burden of hospital related infections. Our findings highlight the growing incidence of carbapenem‐resistant *Acinetobacter spp*. making it one of the most difficult to treat pathogens. A large prevalence of carbapenem‐resistance has been reported for *P. aeruginosa* and *A. baumannii* across the Arab League, reaching 50% and 88% of isolates in some countries as suggested by Moghnieh et al. [21]. *Enterobacter spp*. displayed low susceptibility towards aztreonam, cefepime, and piperacillin/tazobactam, with susceptibilities of 61%, 68%, and 49%, respectively. Similarly, *K. pneumonia* demonstrated high resistance towards carbapenems suggesting the rise of carbapenemase‐resistant *K. pneumonia* aligning with the emergence of carbapenem‐resistance across several countries such as Egypt and Syria [21]. *K. pneumonia* and *E. coli* have both demonstrated limited sensitivity to third‐generation cephalosporins, as illustrated in Table 2. This pattern is consistent with findings from countries in the East, such as Palestine and Syria, where over 50% of tested isolates were resistant to third‐generation cephalosporins. Similarly, resistance rates of around 30%–50% were observed in isolates from Jordan and Lebanon [21].

To combat antibiotic resistance, implementing effective antimicrobial stewardship programs is essential to ensure antibiotics are used only when necessary and to minimize inappropriate use. Infection control practices, such as hand hygiene, contact precautions, and personal protective equipment, are critical in preventing the spread of resistant bacteria. The use of rapid diagnostic tools should be prioritized to enable timely pathogen identification, allowing for precise therapy and reducing reliance on broad‐spectrum antibiotics. Additionally, expanding research to understand prescribing patterns and refining stewardship goals in specialized settings, such as pediatric oncology, will enhance resistance management. Educational initiatives for healthcare workers are equally vital to raise awareness about the judicious use of antibiotics and the growing challenge of resistance.

This study was subject to certain limitations. Firstly, it is a retrospective, single‐center investigation. Moreover, the absence of a non‐cancer control group limits the generalizability of our findings to broader pediatric populations. The identification of true bacterial infections may be challenging, and there is a possibility of including colonizing microbes that might not be associated with clinically significant infections. This limitation is attributed to the focus on microbiologically confirmed cultures rather than clinically defined infections, as clinical signs and symptoms were not incorporated into the enrollment criteria. Additionally, the inclusion of only episode‐based isolates within a 30‐day interval might have resulted in the omission of some additional relevant positive cultures. Nevertheless, our comprehensive approach involved analyzing positive cultures from diverse specimen types, steering away from a narrow focus on a specific site such as the bloodstream. Additionally, we studied the pattern of resistance over 8 years for pathogens, providing a long‐term perspective on resistance trends. In settings lacking local epidemiological data, our results hold practical value for refining prophylactic and treatment strategies across pediatric patient cohorts. We encourage future research to evaluate mortality rates associated with multidrug‐resistant infections, as these are critical for understanding the impact of resistant pathogens in clinical settings.

## 5 Conclusion

Overall, our study has provided an assessment of prevalent pathogens along with their resistance profiles among pediatric patients with cancer showing that Gram‐negative pathogens have prevailed. This underscores the importance of ongoing local surveillance for infections in pediatrics and the implementation of antibiotic stewardship in pediatric oncology. This study also highlights the changes in resistance patterns throughout the years with MRSA and *P. aeruginosa* showing a declining trend, emphasizing the need for continuous assessment of the type of bacterial pathogens to better guide choices of antimicrobials.

## Author Contributions

**Dana Hassouneh, Razan Zatarah, Aseel AbuSara, Lama Nazer, and Iyad Sultan:** were involved in the conceptualization, methodology, data validation, and analysis of the study. **Amal Abu Ghosh:** contributed to the conceptualization and methodology. **Haitham Al Aryan:** was responsible for data collection.

## Conflicts of Interest

The authors declare no conflicts of interest.

## Data Availability

The data that support the findings of this study are available on request from the corresponding author. The data are not publicly available due to privacy or ethical restrictions.
